# Transbronchial Lung Cryobiopsy (TBLC) in an Acute Respiratory Distress Syndrome (ARDS) Patient Under Extracorporeal Membrane Oxygenation (ECMO)

**DOI:** 10.7759/cureus.81907

**Published:** 2025-04-08

**Authors:** Pierre Caro, Pascal Schlossmacher

**Affiliations:** 1 Intensive Care Unit, Université de La Réunion, Saint-Denis, FRA; 2 Pulmonology Department, Université de La Réunion, Saint-Denis, FRA

**Keywords:** ards, extracorporeal membrane oxygenation, intensive care, surgical lung biopsy, transbronchial lung cryobiopsy

## Abstract

We report one of the first known cases of transbronchial lung cryobiopsy (TBLC) performed in a patient with severe acute respiratory distress syndrome (ARDS) receiving extracorporeal membrane oxygenation (ECMO). After transient stopping of anticoagulation, TBLCs were performed in a controlled environment at the bedside in the intensive care unit (ICU). The ECMO avoided severe oxygenation deterioration, and bleeding was controlled by Fogarty balloon inflation. No major complications occurred, and the pulmonary biopsies helped obtain prognostic information and decide the most appropriate management. Nevertheless, the safety of this technique in such high-risk patients should be further investigated in larger case series, as it can be harmful if not performed in an appropriate environment.

## Introduction

Acute respiratory distress syndrome (ARDS) is characterized by acute onset of bilateral pulmonary infiltration and severe hypoxemia in the absence of cardiogenic pulmonary edema. It is a severe and widespread condition, affecting up to 79 cases per 100,000 individuals per year, with high morbidity and mortality rates ranging from 40% to 60% [1]. The etiology of ARDS is diverse, but it is frequently associated with sepsis (bacteremia, pneumonia), trauma, broncho-aspiration, pancreatitis, burns, and even blood transfusions. Diffuse alveolar damage (DAD) is typically observed in pathological analyses of lung specimens; however, other findings may also be present, such as underlying interstitial lung disease, pneumonia, cancer, organizing pneumonia, or alveolar hemorrhage [1]. The aforementioned conditions do not all respond equally to initial corticosteroid treatment, which remains a topic of ongoing debate and controversy [2].

Pathological samples obtained through standard diagnostic tools and techniques, including bronchoalveolar lavage (BAL), transbronchial forceps biopsies (TBB), and open lung biopsy, differ in both diagnostic yield and safety profile. Complications can range from transient oxygenation deterioration to pneumothorax and massive hemorrhage [3,4]. Consequently, there is currently no definitive recommendation on the optimal diagnostic procedure to employ after initial examinations have been conducted. Lung biopsies are performed selectively, particularly when alternative causes are suspected, as these may necessitate timely and specific clinical management and treatment.

Transbronchial lung cryobiopsy (TBLC) is a relatively recent biopsy technique that utilizes carbon dioxide to freeze soft tissue. The primary advantage of TBLC is that it yields larger histological samples than traditional TBB while causing fewer complications than those associated with surgical lung biopsy [4].

TBLCs are particularly useful for diagnosing diffuse parenchymal lung disease, with minimal risks when performed in experienced centers [5-8]. Recently, Dincer et al. published a series of TBLCs conducted in five ARDS cases admitted to an intensive care unit (ICU), reporting no major complications [9].

## Case presentation

**Figure 1:**
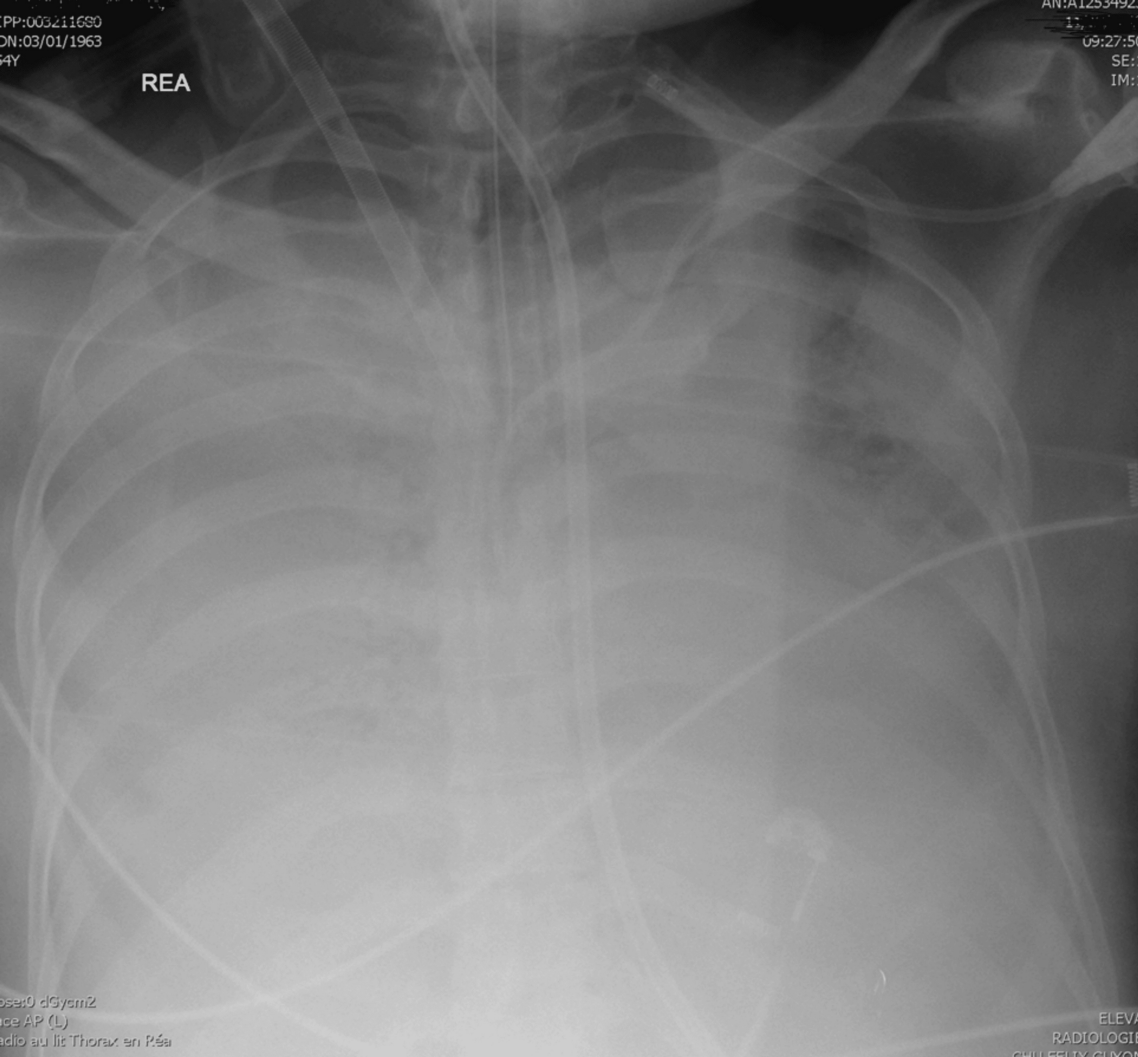
Chest X-ray at the bedside Diffuse alveolar shadows (acute respiratory distress syndrome, ARDS)

A 55-year-old male patient, without significant comorbidities, was admitted to the ICU with ARDS complicating an A-type influenza infection, confirmed by polymerase chain reaction performed on a BAL. The patient’s respiratory condition rapidly deteriorated, and despite the use of paralyzing agents, prone positioning, and completion of a full course of steroids, extracorporeal membrane oxygenation (ECMO) had to be initiated, along with broad-spectrum antibiotics, on day eight. As no significant improvement was observed by day 16 - with persistent elevated oxygen requirements, decreased pulmonary compliance, and unchanged radiological findings (Figure 1) - a multidisciplinary discussion led to the decision to perform TBLCs. The purpose of the biopsies was to rule out an unidentified opportunistic infection, assess for features of organizing pneumonia, or confirm lesions of diffuse alveolar damage, even at a fibrotic stage.

**Figure 2:**
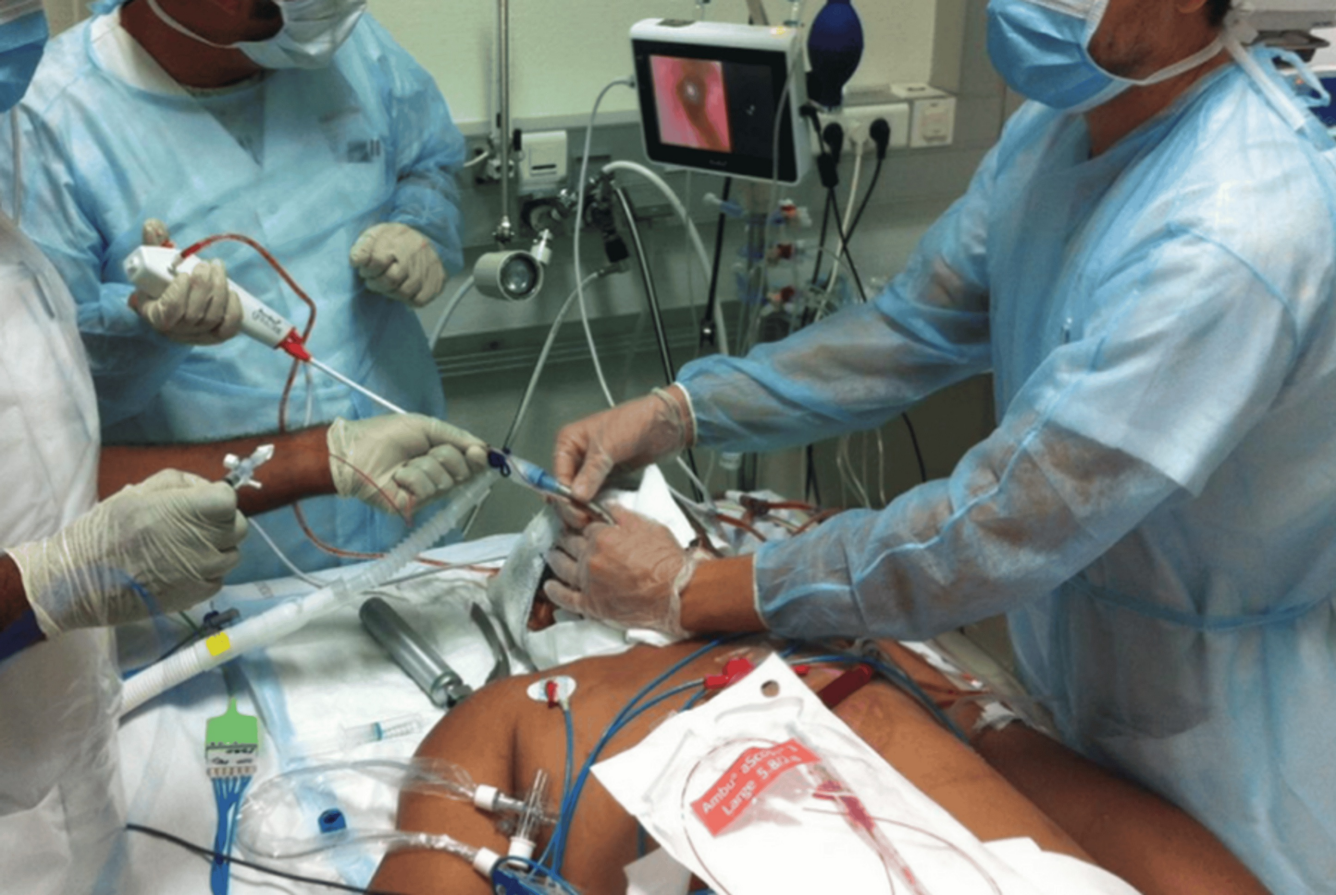
Photography showing transbronchial lung cryobiopsy (TBLC) procedure under extracorporeal membrane oxygenation (ECMO) at the bedside The bronchoscopist controls Fogarty balloon’s inflation after each cryobiopsy. The first assistant kept the balloon inflated in place and deflated it under direct bronchoscopic vision. In cases of bleeding, the balloon is re inflated. The second assistant managed patient’s monitoring and ECMO.

Prior to the procedure, anticoagulation was temporarily discontinued. TBLCs were performed in a controlled ICU environment, with the patient paralyzed, sedated, and under ECMO (blood flow: 5 L x min^-1^, expired oxygen fraction (FiO₂): 100%, fresh gas flow: 9 L x min⁻¹, femoral-jugular venous access). The procedure was conducted at the bedside by an interventional respiratory physician (Figure 2). A 1.9-mm ERBECRYO® 2 cryoprobe (Erbe Elektromedizin GmbH, Tübingen, Germany) was used. Tracheal intubation was performed with a specialized device that permitted the placement of a Fogarty balloon in a separate lumen (7.5 mm inner diameter; Rüsch GmbH, Germany), and ventilation was maintained via a closed circuit. Fluoroscopic guidance was unavailable.

**Figure 3:**
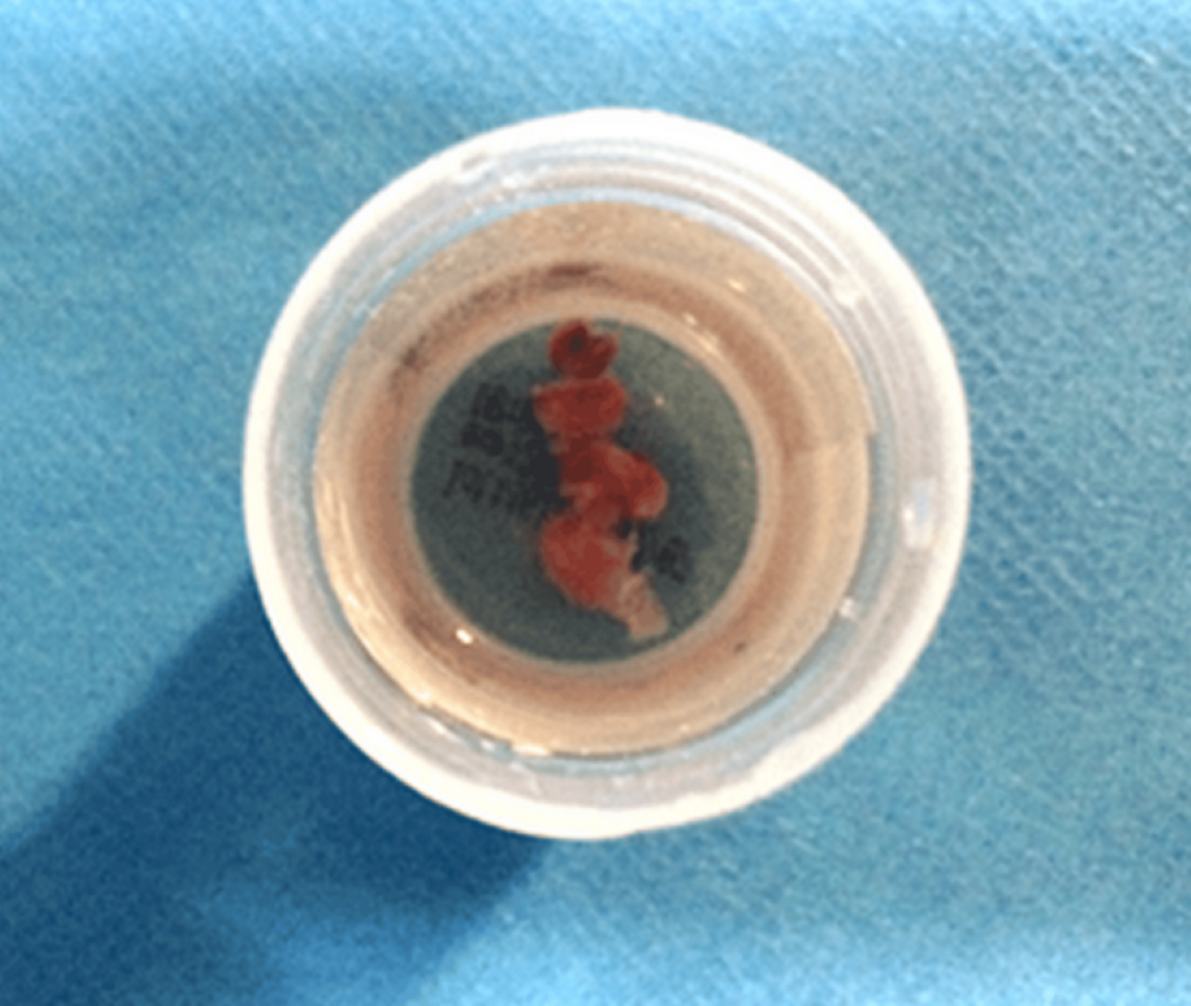
Macroscopic view of the lung tissue samples obtained by transbronchial lung cryobiopsy (TBLC)

The probe was blindly advanced into the distal lung parenchyma until resistance was encountered at a subpleural location, at which point it was withdrawn by 1-2 cm. Four samples were collected: two from two different segments of the same lobe, following an initial freezing time of 8 s (sizes ranging from 6 to 9 mm, with surface areas of 30-54 mm²) (Figure 3). To prevent uncontrolled bleeding, an angioplasty balloon (Fogarty balloon, size 4) was systematically inflated after each biopsy, as recommended [9,10]. Only mild bleeding was observed, lasting less than five minutes and controlled without the need for cold saline instillation. The procedure followed the most recent guidelines [11-13].

**Figure 4:**
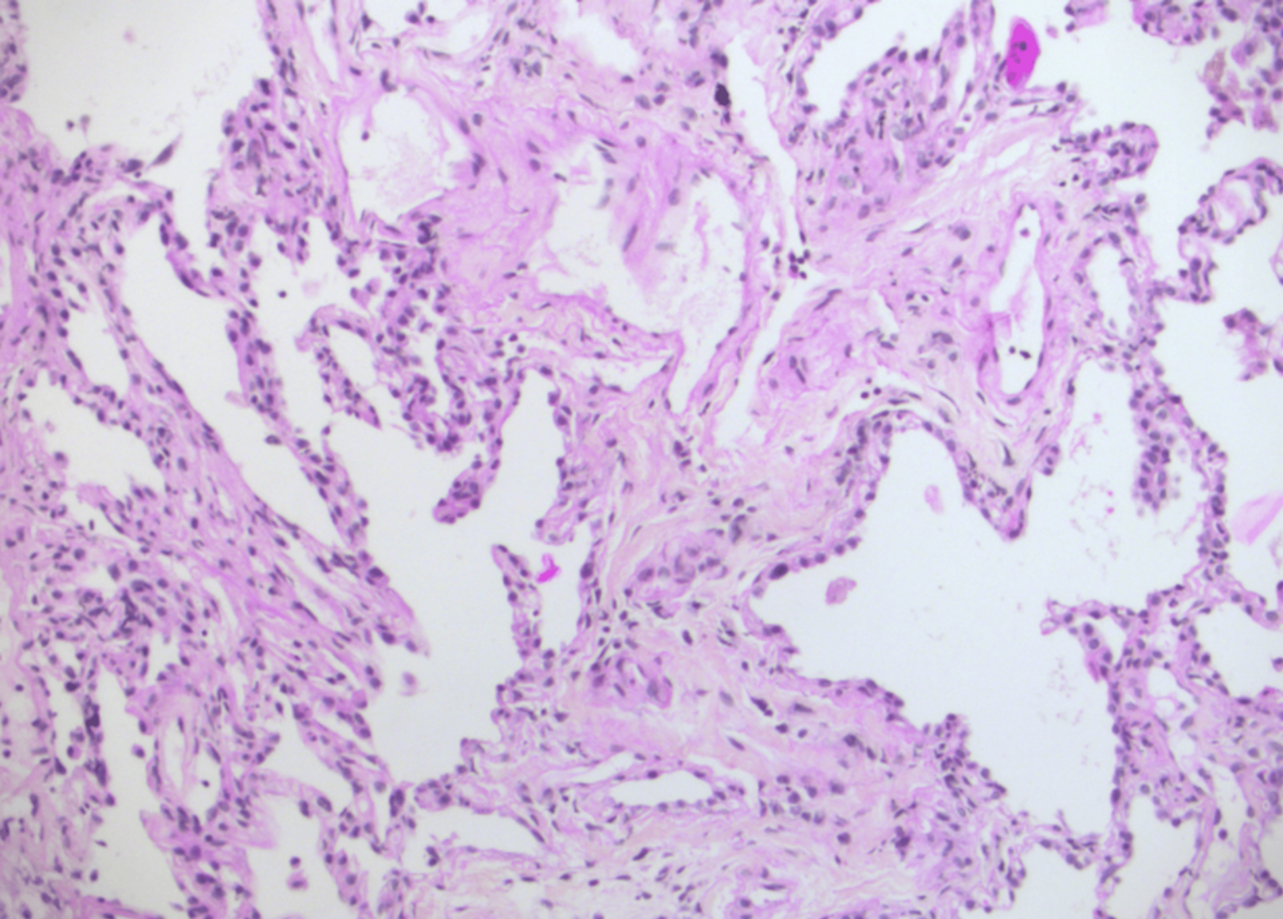
Microscopic view of the lung tissue samples obtained by transbronchial lung cryobiopsy (TBLC) X200 (HPS stain) Pulmonary fibrosis with thickened alveolar walls

**Figure 5:**
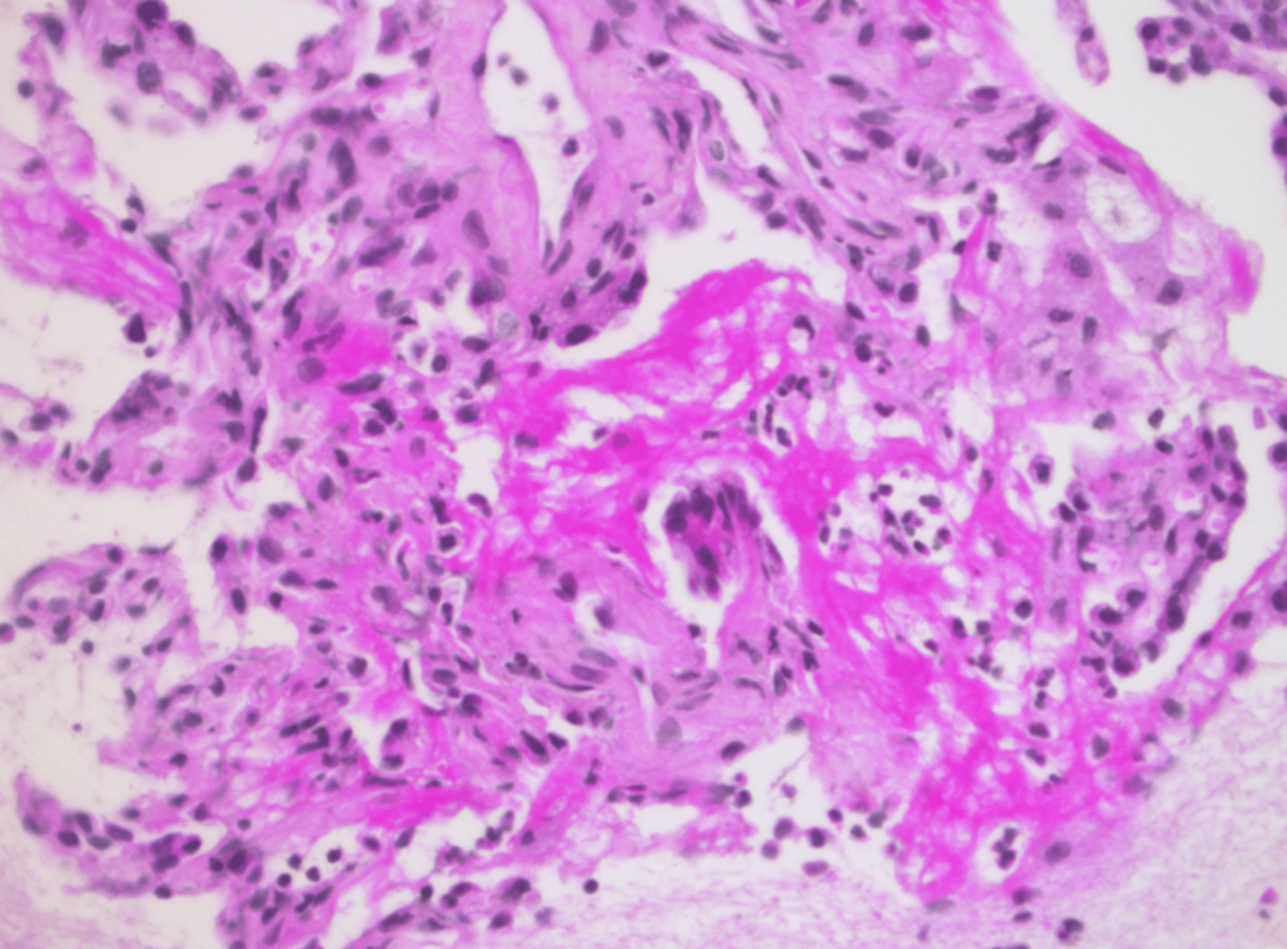
Microscopic view of the lung tissue samples X400 (HPS stain) Alveolar edema and hyaline membranes (diffuse alveolar damage at fibrotic stage)

No other complications (e.g., pneumothorax) were reported. The collected samples demonstrated DAD with extensive interstitial fibrosis (Figures 4-5). Given the poor prognosis associated with these findings, active supportive treatments were withdrawn, and the patient passed away shortly thereafter.

## Discussion

Following the recent publication of the first series of TBLCs performed in ICU patients with ARDS [9], we report the first case of TBLC conducted in a patient receiving ECMO. The most recent recommendations and insights from previous cases [11-13] were applied, and a Fogarty balloon was utilized to control potential severe bleeding during the biopsies. The value of pulmonary biopsies is inherently dependent on the quality and quantity of the samples obtained, balanced against the associated risks. At our center, TBLCs are routinely performed for the diagnosis of interstitial lung diseases to reduce complications compared to surgical biopsies.

Performing TBLCs in this case was particularly challenging. Firstly, the patient had severe ARDS, and the safety of performing TBLCs in such a condition remains uncertain. To date, only Dincer et al., in their series of TBLCs performed in five ARDS patients, reported no significant bleeding or pneumothorax [9]. However, we believe that the risk of complications could be higher in this context. Secondly, our patient was receiving ECMO, which may further increase the risk of bleeding. Indeed, studies have demonstrated that patients on ECMO have a significantly higher incidence of hemorrhagic complications [1]. Conversely, the use of ECMO in severe ARDS provides the advantage of maintaining optimal oxygenation throughout the procedure, thereby reducing the risk of severe hypoxemia that could arise if TBLCs were performed under conventional mechanical ventilation alone. Fortunately, no major complications occurred, and the biopsies provided valuable prognostic information, enabling the most appropriate management decisions.

## Conclusions

We report that TBLC can be performed in a critically ill patient with ARDS receiving ECMO. However, the safety of this technique in such high-risk patients requires further investigation through larger case series, as it may be harmful if not conducted in an appropriate and controlled environment. Given the potential risks, a standardized protocol and experienced proceduralists are essential to minimize complications and improve patient outcomes.
